# Bone matrix development in steroid-induced osteoporosis is associated with a consistently reduced fibrillar stiffness linked to altered bone mineral quality

**DOI:** 10.1016/j.actbio.2018.05.053

**Published:** 2018-08

**Authors:** L. Xi, P. De Falco, E. Barbieri, A. Karunaratne, L. Bentley, C.T. Esapa, N.J. Terrill, S.D.M. Brown, R.D. Cox, G.R. Davis, N.M. Pugno, R.V. Thakker, H.S. Gupta

**Affiliations:** a School of Engineering and Material Sciences, Queen Mary University of London, London E1 4NS, UK; b Department of Nuclear Engineering, North Carolina State University, Raleigh, NC 27607, USA; c Department of Mechanical Engineering, University of Moratuwa, Sri Lanka; d MRC Mammalian Genetics Unit and Mary Lyon Centre, MRC Harwell, Harwell Science and Innovation Campus, OX11 0RD, UK; e Academic Endocrine Unit, Nuffield Department of Clinical Medicine, Oxford Centre for Diabetes, Endocrinology and Metabolism (OCDEM), University of Oxford, Churchill Hospital, Headington, Oxford OX3 7JL, UK; f Beamline I22, Diamond Light Source Ltd., Diamond House, Harwell Science and Innovation Campus, Chilton, Didcot, Oxfordshire OX11 0DE, UK; g Queen Mary University of London, Barts and the London School of Medicine and Dentistry, Institute of Dentistry, E1 2AD, UK; h Department of Biomaterials, Max Planck Institute of Colloids and Interfaces, D-14424 Potsdam-Golm, Germany; i Laboratory of Bio-Inspired & Graphene Nanomechanics, Department of Civil, Environmental and Mechanical Engineering, University of Trento, Via Mesiano, 77, 38123 Trento, Italy; j Ket Lab, Edoardo Amaldi Foundation, Italian Space Agency, Via del Politecnico snc, 00133 Rome, Italy; k Department of Mathematical Science and Advanced Technology (MAT), Yokohama Institute for Earth Sciences (YES) 3173-25, Showa-machi, Kanazawa-ku, Yokohama-city, Japan

**Keywords:** Glucocorticoid induced osteoporosis, Synchrotron X-ray nanomechanical imaging, Nanoscale deformation mechanisms, Multiscale mechanical modelling

## Abstract

Glucocorticoid-induced osteoporosis (GIOP) is a major secondary form of osteoporosis, with the fracture risk significantly elevated – at similar levels of bone mineral density – in patients taking glucocorticoids compared with non-users. The adverse bone structural changes at multiple hierarchical levels in GIOP, and their mechanistic consequences leading to reduced load-bearing capacity, are not clearly understood. Here we combine experimental X-ray nanoscale mechanical imaging with analytical modelling of the bone matrix mechanics to determine mechanisms causing bone material quality deterioration during development of GIOP. *In situ* synchrotron small-angle X-ray diffraction combined with tensile testing was used to measure nanoscale deformation mechanisms in a murine model of GIOP, due to a corticotrophin-releasing hormone promoter mutation, at multiple ages (8-, 12-, 24- and 36 weeks), complemented by quantitative micro-computed tomography and backscattered electron imaging to determine mineral concentrations. We develop a two-level hierarchical model of the bone matrix (mineralized fibril and lamella) to predict fibrillar mechanical response as a function of architectural parameters of the mineralized matrix. The fibrillar elastic modulus of GIOP-bone is lower than healthy bone throughout development, and nearly constant in time, in contrast to the progressively increasing stiffness in healthy bone. The lower mineral platelet aspect ratio value for GIOP compared to healthy bone in the multiscale model can explain the fibrillar deformation. Consistent with this result, independent measurement of mineral platelet lengths from wide-angle X-ray diffraction finds a shorter mineral platelet length in GIOP. Our results show how lowered mineralization combined with altered mineral nanostructure in GIOP leads to lowered mechanical competence.

**Significance Statement:**

Increased fragility in musculoskeletal disorders like osteoporosis are believed to arise due to alterations in bone structure at multiple length-scales from the organ down to the supramolecular-level, where collagen molecules and elongated mineral nanoparticles form stiff fibrils. However, the nature of these molecular-level alterations are not known. Here we used X-ray scattering to determine both how bone fibrils deform in secondary osteoporosis, as well as how the fibril orientation and mineral nanoparticle structure changes. We found that osteoporotic fibrils become less stiff both because the mineral nanoparticles became shorter and less efficient at transferring load from collagen, and because the fibrils are more randomly oriented. These results will help in the design of new composite musculoskeletal implants for bone repair.

## Graphical abstract

**Figure:**
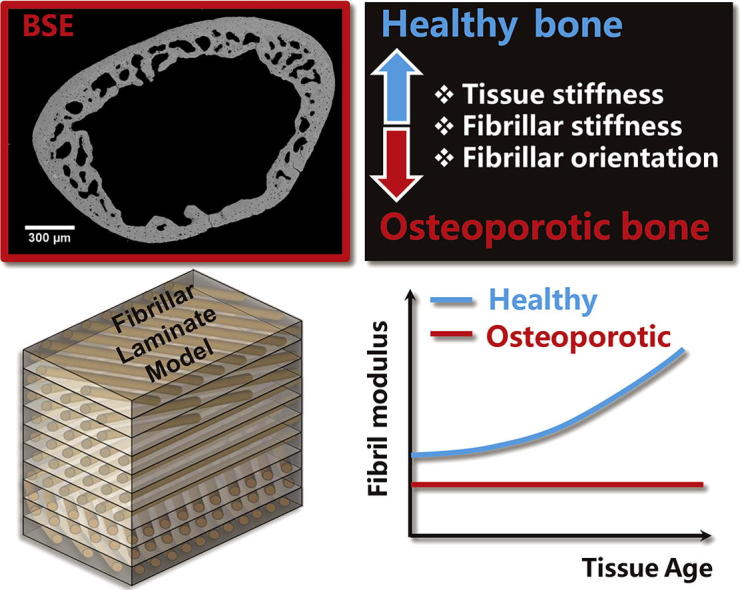

## 1 Introduction

The reduced mechanical and structural competence of bone in musculoskeletal disorders (e.g. osteoporosis and osteoarthritis) arise from both alterations in the mineralization dynamics (*via* altered cellular activity) as well as intrinsic changes in the mineralized collagen matrix (as in disorders like osteogenesis imperfecta), but the mechanisms linking matrix alterations to reduced functionality are not always clear [1], [2], [3], [4]. Glucocorticoid-induced osteoporosis (GIOP) is one of such disorders, affects 1–3% of the general population and is characterised by a rapid increase in fracture risk in patients taking anti-inflammatory steroidal medications (glucocorticoids or GCs) [5], [6], [7], [8]. About 30 to 50 percent of patients with chronic glucocorticoid treatment suffer from osteoporotic fractures [9]. In cancellous bone, GCs can cause suppression of bone formation and enhanced and prolonged resorption through direct effects on osteoblasts, osteoclasts and osteocytes [10], [11]. As a result, in GIOP, bone resorption (osteoclastic activity) is not matched by bone formation (osteoblastic activity), which results in reduced bone mass. Most notably, however, the increase in fracture risk in GIOP is larger than predicted from changes in bone mass alone (relative to healthy bone) [12], implying that changes in the bone matrix and microarchitecture play an important role. For example, increased vertebral fracture risk in GIOP-patients is associated with trabecular thinning (microarchitecture), and material-level changes have been observed as well in cancellous bone [13], [14], [15]. Alterations in bone metabolism – mainly affecting cancellous bone – in terms of cellular changes have been recognized in GIOP [1], [16]. Therefore, understanding the structural mechanisms in bone tissue – at multiple hierarchical levels – which cause the reduction in mechanical properties in GIOP would thus be of considerable clinical relevance. Such an understanding would also shed light on the mechanical relevance of bone matrix quality [3], [4], [17] in a prototypical example of secondary osteoporosis, where the current gold standard method of assessment of osteoporotic fracture risk – bone mineral density or BMD [18], [19], [20] – is inadequate for fracture risk predictions [5], [12], [21].

The cellular mechanisms of action of GIOP include disruption of the *Wnt*/LRP5 signalling pathway, leading to reduction of new bone formation, *via* osteoblastic suppression and increased osteoblast apoptosis [12], [22]. Further, GC action prolongs osteoclast lifespan, which leads to greater bone resorption [11]. More recently, researchers have focused on the alteration in osteocyte metabolism, which leads to local demineralization around lacunae [23], [24]. However, the structural mechanisms by which these cellular-level changes reduce mechanical competence remains to be elucidated. In postmenopausal women treated with GCs, reduced bone formation led to trabecular thinning [14], [15], and an increased fracture risk when compared to untreated patients with similar bone mineral density (BMD) [12]. At the microscale, alteration of the resorption cavities in GIOP from rounded to elongated shapes have been shown, *via* finite element modelling, to explain in part the stiffness reduction in GIOP [25]. The use of quantitative computed tomography – rather than DXA-based BMD measurements – was found to better explain male vertebral fracture in GIOP [26], further highlighting the importance of micro-architectural changes in bone quality. Studies using micro computed tomography (micro-CT) showed that GCs treatment results in a reduced number, volume and connectivity of trabeculae and reduced cortical thickness in patients [13], [27]. GCs treated mice showed larger osteocyte lacunae surrounded by “halos” of hypomineralized bone tissue with ∼30–40% reduction in local bone mineralization compared to normal mice, as well as loss of trabecular bone volume [13], [28].

At the level of the mineralized bone matrix (“material” level [3], [4]), however, relatively few studies exist, and the link between the growth and development of an altered bone matrix and the change in material-level mechanics is unexplored. Material-level changes in bone matrix quality – whether *via* increased collagen crosslinking in ageing human bone [29], reduced extrafibrillar mineralization in rickets [30], or *via* actions of anti-osteoporotic drugs [31] have been demonstrated in several different bone pathologies, and it is hence likely that GIOP-bone is associated with such changes as well. In this regard, a few studies have shown that the stiffness of the bone matrix also reduces in GIOP [13], [32]. For example, localized atomic force microscopy and nanoindentation data on mice bone show that tissue regions around osteocytes have both lower mineralization (hypomineralized “haloes”) as well as elastic modulus [13]. Our group explored the link between such mechanical changes and structural deformation mechanisms at the fibrillar level [32] using *in situ* X-ray nanomechanics. We used synchrotron small-angle X-ray diffraction (SAXD) to measure the deformation of the mineralized collagen fibrils in GIOP bone, and found reductions in fibrillar-level moduli and increased randomness in fibrillar orientation at a single age-point in a murine model of GIOP. The model arises from a corticotrophin-releasing hormone (*Crh*) promoter mutation that was associated with elevated plasma and urinary concentrations of corticosterone in mutant heterozygous mice (*Crh^−120/+^*) when compared to wild-type (WT) mice (*Crh^+/+^)* [32], [33]. Most recently, Fowler and co-workers have also shown that glucocorticoid-treated mice exhibited a disorganized fibrillar structure and matrix hypermineralization in murine subchondral bone [34]. Despite these recent experimental advances, both (a) the developmental- and age-related changes in bone matrix quality in GIOP and (b) structural models of the alterations in the mineralized collagen nanostructure leading to such reductions in mechanics have been very little studied.

In this study, we hypothesize that GIOP leads to a consistent reduction of the nanoscale fibrillar mechanics of bone throughout development, and that alterations in the mineralized fibrillar architecture – beyond overall mineral content – can be implicated in these reductions. Combining *in situ* X-ray nanomechanical imaging using SAXD, microcomputed tomography, micromechanical modelling and wide-angle X-ray diffraction of the mineral crystallite phase, we quantify the changes in bone matrix quality at multiple age points during tissue development in a *Crh^−120/+^* mice, which are a model of Cushing’s syndrome. We use Cushing’s syndrome (endogenous production of corticosteroids) as a model for GIOP, as there is similar fracture risk associated with endogenous production and exogenous administration of corticosteroids [16]. The aim of the study was to clarify whether the changes in bone matrix nanomechanics in GIOP are continuous or discontinuous with tissue maturation, and how the altered mechanical properties could be interpreted *via* a structural model of the mineralized fibril arrays at the nano- and micro-level.

## 2 Materials and Methods

### 2.1 Animals

Bone tissue from female *Crh*^−120/+^ mice on a C57BL/6 genetic background (3rd generation) and *Crh^+/+^* littermates aged 8, 12, 24 and 36 weeks were studied. Mouse samples were stored at −20 °C before experiments. The mice were bred as part of a prior study [33], where all animal studies were carried out using guidelines issued by the UK Medical Research Council, in Responsibility in Use of Animals for Medical Research (July 1993) and Home Office Project License numbers 30/2433 and 30/2642.

### 2.2 Sample preparation for *in-situ* tensile testing

**Fig. 1:**
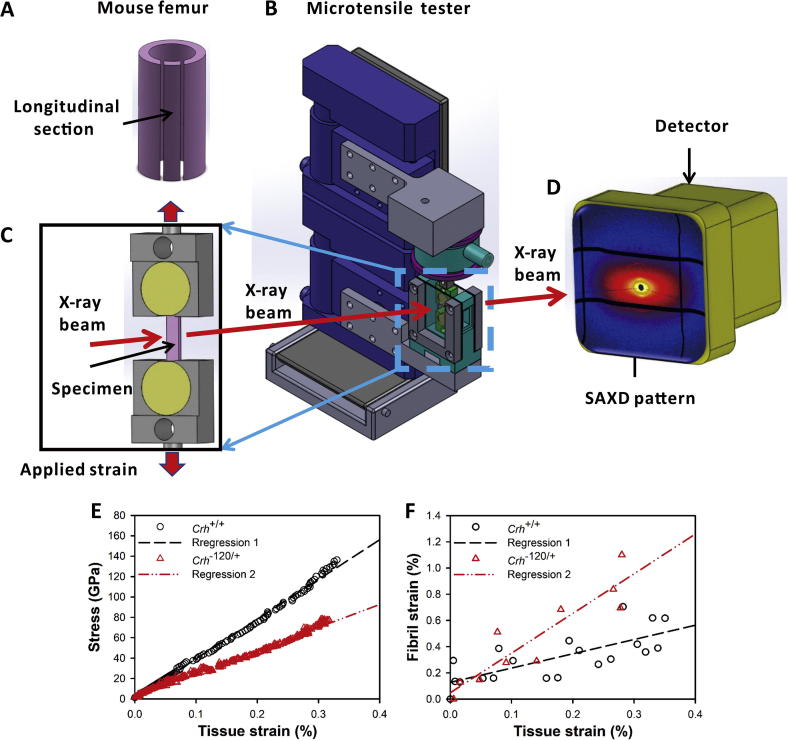
Experimental configuration for *in situ* nanomechanics with synchrotron SAXD: (A) Schematic of mouse femur, with tensile test specimen sectioned along the long axis of femur. (B) Tensile tester with bone sample mounted along the X-ray beam path in transmission geometry. (C) Magnified view of sample and tensile grips in fluid chamber, with tensile strain along the vertical direction. (D) Si pixel detector used for acquiring SAXD pattern from incident X-ray beam interacting with bone sample. (E) Typical stress - tissue strain and (F) fibril strain – tissue strain plots for femoral mid-shaft of *Crh^+/+^* and Crh*^−120/+^* mice aged 36 weeks. Straight lines indicate linear regression (R^2^ = 0.55 for *Crh^+/+^* (dashed) and R^2^ = 0.84 for Crh*^−120/+^* (dash-double dot)).

Bone samples were prepared for *in situ* microtensile testing, as described previously [30]. Longitudinal sections of murine femora were prepared using a water-irrigated low speed diamond saw (Fig. 1A). The distal and proximal ends of anterior femora strips were secured in dental ionomer such that samples could be mounted in the microtensile tester (Fig. 1B, C). The dental ionomer was treated for 20s with UV light exposure while the mid-diaphysis of bone was covered by lead tape during UV light exposure to prevent any UV-induced tissue alteration. The typical gauge length of bone samples was ∼5–6 mm in length, ∼1 mm in width and ∼0.2 mm in thickness. The total length of the femora were ∼9–10 mm with 4 mm of the bone distal and proximal ends embedded in dental ionomer. Post-embedding, Vernier calipers were used to carefully measure the distance between dental ionomer moulds to determine gauge length for each sample. Samples were then wrapped in PBS-soaked tissue paper and stored at −20 °C before used for mechanical testing. Number of samples tested at 8, 12, 24 and 36 weeks were 5, 9, 7 and 7, respectively, for *Crh^+/+^* mice; and 6, 10, 6, and 4, respectively, for *Crh*^−120/+^ mice. The unequal number of samples are because some samples were broken or otherwise damaged while mounting in the tester during the beamtime.

### 2.3 *In-situ* microtensile testing with synchrotron X-Ray scattering and diffraction

*In-situ* tensile testing combined with real time synchrotron SAXD were used to measure macroscopic tissue strain ε_t_ (from non-contact video extensometry) and fibril strain ε_f_ (from SAXD frames) concurrently, as reported by Gupta et al. [35]. The meridional stagger D of collagen molecules in bone fibrils leads to an axial diffraction pattern in the SAXD region of reciprocal space. The third order meridional collagen reflection was used to measure the D-period. The fibril strain ε_f_ was calculated from the percentage increases in D-period during tensile testing of bone [29], [35], [36].

Samples were loaded uniaxially in tension using a customized microtensile tester (Fig. 1B) which was mounted on a two-axis motorized stage at Beamline I22, Diamond Light Source, UK. Samples were loaded in a fluid chamber, which provided a physiological environment (PBS solution, room temperature) for the bone. Motor strain was measured using a DC linear-encoder stage (M112.1DG; Physic Instruments, UK) and load data was recorded using a tension/compression load cell (model SLC31/00025 with 111 N range; RDP Electronics Ltd, UK). A waterproof marker with 0.05 mm tip from a pen (Copic multiliner, Too Corporation, Japan) was used to make optical markers on the bone mid-diaphysis and a Basler Ace USB Camera (MultiPix Imaging Components Ltd, UK) was used to acquire consecutive images of samples with frame rate of 5 fps during tensile loading. Tissue strain ε_t_ was measured as percent increase of distance between two horizontal optical markers on the bone during loading. Custom software written in LabVIEW 2013 (National Instruments, UK) was used to control the DC linear-encoder stage, load cell and camera such that motor strain, load data and images were collected concurrently at 0.2 s intervals during mechanical loading. Samples were loaded to failure using a constant velocity of 0.002 mm/s, corresponding to a strain rate of 0.0004 s^−1^, used in our previous studies [30], [32], [37], [38].

A synchrotron X-ray beam with wavelength of 0.8857 Å and beam cross section of ∼ 10 μm × 10 μm at the sample was used to measure the SAXD and WAXD patterns. The patterns were acquired using a Pilatus P3-2 M detector [39] (Fig. 1D) with pixel resolution of 1475 × 1679 pixels and pixel size of 172 × 172 μm^2^. The sample-to-detector distance for SAXD patterns was ∼1000 mm as measured with a silver behenate standard. The sample-to-detector distance for WAXD patterns was ∼175 mm as measured with a silicon standard. A SAXD pattern with an exposure time of 0.5 s was collected every 10 s during tensile loading up to failure. In order to minimize the absorption of X-ray scattering in the fluid, the physiological fluid chamber was designed with ∼4 mm total X-ray path length in the PBS solution. To reduce the effects of X-ray radiation on the bone mechanical properties, the X-ray shutter was closed between exposures [40].

### 2.4 Small angle X-ray scattering (SAXS) and diffraction (SAXD) data analysis

2D SAXD patterns of mice bones acquired during tensile testing were used for calculation of fibril D-period and fibril strain ε_f_. The data analysis software package DAWN [41], [42] (www.dawnsci.org) was used in conjunction with a customised Python script for SAXD data analysis. 2D SAXD frames were radially integrated over 20° angular sectors centred along the loading direction to get 1D intensity profiles around the third-order reflection of collagen. Then, the obtained 1D profiles were automatically fitted using a custom script, which was added as a Python XY-to-XY operation to the *Processing* pipeline in DAWN [42]. A Gaussian peak combined with a linear background term were used for the peak fitting of the third-order reflection from collagen fibrils. The obtained peak positions were used to calculate the meridional stagger D-periods of collagen fibrils, and fibril strains were measured from the percentage changes of collagen D-periods during tensile loading relative to the unstrained state. The effective fibril modulus (E_f_ = dσ/dε_f_) was defined as the slope of tissue-level stress versus fibril strain from the elastic region of deformation, following prior work [30], [32].

### 2.5 Determination of degree of fibrillar alignment

Degree of alignment of the mineral crystals (the ρ-parameter) can be determined from angular SAXD intensity profiles following previous work [43], [44], [45], [46]. The intensity distribution obtained from azimuthal integration of the SAXD pattern shows two peaks separated by 180°. Parallel-aligned particles contribute to the area under the peaks, whereas randomly oriented particles contribute to the area under the constant background. The ρ-parameter is defined as ρ = (area under the peaks)/(total area) [43], [44], [45], [46]. Perfectly-parallel aligned mineral platelets will give a narrow streak perpendicular to the long axis of the mineral particles on the SAXD pattern giving a ρ-parameter of 1, randomly oriented particles will give a spherical SAXD pattern (ρ = 0), and partially aligned mineral crystals will give an elliptical SAXD pattern (0 < ρ < 1). In this work, the mineral platelets will be taken as parallel to the collagen fibrils, as per previous work [47], and hence the ρ-parameter can be taken as a proxy for the degree of collagen fibrillar alignment. Due to a relatively lower SAXD signal intensity for the azimuthal profile of the meridional reflections, it was decided to use the higher-intensity diffuse scattering angular SAXS profile for fibrillar alignment, in preference to the full width at half maximum of the angular meridional SAXD profile used earlier [32].

### 2.6 Wide angle X-ray diffraction (WAXD) data analysis

The crystallographic structure of the mineral particles in bone consist of hexagonal cubic apatite with the c-axis predominantly oriented along the fibril direction [48]. The lattice spacing was calculated from the (0 0 2) peak centre position of apatite along the vertical direction in the WAXD region of reciprocal space [35]. A Lorentzian function was used to fit the peak and obtain the full width at half maximum (FWHM) of the (0 0 2) reflection (Figure S2, Supplementary Information). The mean crystallite length of the mineral phase in bone was calculated by Scherrer’s equation [45], [49]: L = kλ/B cos θ, where L is the mean mineral crystal length; k is a constant related to the crystallite shape and falls in the range 0.87–1.0; λ is the wavelength of the X-rays (0.8857 Å). B is the FWHM of the (0 0 2) reflection of hydroxyapatite (HA) from wide-angle X-ray diffraction (WAXD) patterns, and θ is the Bragg angle of the (0 0 2) reflection.

The broadening of the Debye-Scherrer width (0 0 2) reflection of HA, as measured from WAXD pattern, has two main contributions [45]: B_L_, the crystallite length contribution to the peak broadening, and B_I_, the instrumental broadening contribution. B is expressed as a squared sum function of these contributions: B^2^ = B_L_^2^ + B_I_^2^. The instrumental broadening contribution on the HA crystallite was estimated by calculating the FWHM at a wavevector (*q*) value of (0 0 2) reflection from the linear regression of FWHM - *q* plot for a Si standard. All measured FWHM of (0 0 2) reflection were then corrected with respect to the instrumental broadening contribution to obtain B_L_, which is the mean mineral crystal length (L-parameter).

### 2.7 Backscattered electron imaging

Backscattered electron (BSE) imaging was used to measure the two-dimensional (2D) morphology of the transverse cross section of tibia mid-diaphysis. Three tibias from each age and disease conditions were dehydrated in a graded series of ethanol and xylene and then embedded in a histological embedding poly-methyl-methacrylate (PMMA; three grams of azo-iso-butyronitrile per litre of methyl methacrylate monomer). The PMMA blocks were sectioned using a low-speed diamond saw and polished with grinding papers and diamond liquid suspension with progressively finer grain size down to 1 μm, as previously reported [37], [38]. The final polished surface was a transverse cross section in the mid-diaphysis of tibia. A thin conductive carbon was coated to the samples surfaces before BSE imaging. The BSE imaging was performed with a Scanning Electron Microscope (Inspect F, FEI Ltd, Eindhoven, the Netherlands) equipped with a back-scattering detector. The accelerating voltage was adjusted to be 20 kV and the current to be 160 μA. Sample-to-detector distance was adjusted to be 10 mm for all BSE images. The obtained BSE images had pixel resolution of 1024 × 884 pixels with pixel size of 0.3125 μm. Each BSE image had a range of gray levels from 0 to 255.

### 2.8 X-ray microtomography

Mice femur samples (cut samples already tested by *in situ* microtensile testing) were used again for X-ray microtomography. Three samples at each age point and genotype were used to obtain tomograms and to calculate mean mineral concentration (degree of mineralization) in the tissue at the microscale. X-ray microtomography was performed using a high-definition MuCat scanner [50] equipped with an X-tek ultrafocus X-ray generator (Nikon Metrology, Leuven, Belgium) and Spectral Instruments (Tucson, Arizona, USA) 800 series CCD camera in a time-delay integration readout mode. An accelerating voltage of 40 kV was used for the scanning to obtain tomograms with voxel size of 15 × 15 × 15 μm^3^. The micro‐CT projection data were calibrated to 25 keV monochromatic equivalence and then reconstructed to form a 3D image using a cone-beam back-projection algorithm. The 3D images was converted to a stack of 2D images, and averaged using the Tomview in-house software, which reports the linear attenuation coefficient associated with the gray-level at each voxel in the tomogram. The calculation of the mean mineral concentration in bone using the following relation:

$$\mathrm{Mineral}\;concentration=\frac{\mu }{\mu_{m}}\rho_{m}$$

Here, μ is the measured linear attenuation coefficient and μ_m_ is the mass attenuation coefficient of pure mineral, which is assumed to be calcium hydroxyapatite (μ_m_ = 3.48 cm^2^g^−1^, from the XCOM database [51]). The attenuation from any residual organic component could not be determined but was assumed to be negligible (even in a hydrated state it is much lower than the mineral component). Quantitative analysis of the mean mineral concentration (in units of g/cm^3^) from tomograms were carried out for *Crh^+/+^ mice*, periosteal and endosteal regions of *Crh*^−120/+^ mice with increasing developmental age. The mean mineral concentration is only calculated for the bone tissue, excluding the spaces and voids around and within the sample, by using a gray-level threshold midway between the peaks in the gray-level histogram corresponding to the tissue and corresponding to voids/empty space. Three regions of interest, comprising cortical bone in *Crh^+/+^* femora, periosteal regions away from cavities and endosteal regions near cavities in *Crh*^−120/+^ femora, were used to generate the histogram of mineral concentration (Fig. 4D) using ImageJ software (ImageJ, NIH, USA). The obtained histogram of mineral concentration was used to calculate the weighted mean mineral concentrations, which was plotted against age for three different regions (Fig. 4E,F).

### 2.9 Calculation of microscale porosity

The experimental stress data were post-multiplied by the coefficient 1/(1 − p^3/2^) to incorporate the effects – on the effective cross-sectional area – of a 3D isotropic distribution of internal porosity in bone. In this case the 3D porosity is *p*^3/2^, where *p* is the 2D porosity coefficient. The 2D porosity coefficient *p* is calculated via the following relation:

$$p=\frac{2\text{D}\;\text{area}\;\text{of}\;\text{voids}}{2\text{D}\;\text{area}\;\text{of}\;\text{solid}\;\text{bone}+2\text{D}\;\text{area}\;\text{of}\;\text{voids}}$$

2D area of voids and solid bone was analysed from 2D image slices of X-ray tomographic data using segmentation tools in Avizo software (Visualization Sciences group, Burlington, MA, USA). Gray level based thresholding was manually chosen using Avizo such that the voids and solid bone were completely segmented. The total pixel number located in voids region and solid bone region were used to obtain 2D area of voids and solid bone, respectively (refer to Fig. S1, Supplementary Information (SI) for an example).

### 2.10 Statistical analysis

The variables in our experiment are bone type (*Crh^+/+^* or *Crh*^−120/+^) and age (8, 12, 24 and 36 weeks). To test age-related variation in tissue modulus, and nanoscale parameters like effective fibril modulus, lattice spacing, FWHM, L-parameter, and ρ-parameter, we carried out one-way ANOVA for each bone type as a function of age followed by Tukey's honestly significant difference (HSD) post hoc tests. To compare these same parameters as a function of bone-type at each age, Student’s *t*-tests were used. Lastly, for the sole case of mean mineral concentration, which can be measured from three types of bone tissue (*Crh^+/+^*, *Crh*^−120/+^-ER (endosteal) and *Crh*^−120/+^-PR (periosteal)) at each age, one-way ANOVA tests were performed at each age when three different regions were considered, and *t*-tests when the *Crh*^−120/+^ tissue was considered as a whole.

We note that a two-way ANOVA was not performed (with age and bone-type as variables) due to unequal sample sizes between ages and bone types. Further, to avoid overcrowding the Results figures, the *t*-test indicators of significance are shown via pairwise comparisons, while the more numerous pairwise post-hoc results of age-related variation are summarized in the Results text, and given in tables in the Supplementary Information in full. Excel (Microsoft Office 2016) was used for the Student’s *t*-test and Sigma Plot version 14.0 (Systat Software, San Jose, California) for the ANOVA tests.

### 2.11 Modelling of bone matrix mechanics

**Fig. 2:**
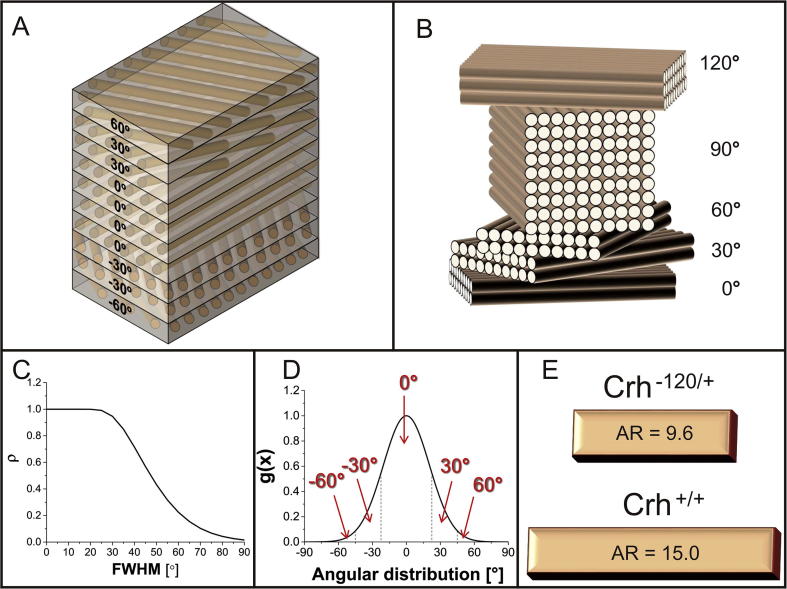
Multiscale model of lamellae of mineralized fibrils: A) Plywood structure modelled as a laminate composite (homogenized plywood structure where each lamella is made of mineralized fibrils). B) Discretization of the rotated plywood model we assumed [58], with a set of lamellae of different thickness which are angularly shifted of 30°. C) ρ-parameter vs FWHM. We assumed (from Fig. 6D) an average value of ρ for the *Crh^+/+^* bone of 0.45 and of 0.3 for the Crh*^−120/+^* bone and determined, respectively, the values FWHM = 48° and 57°. D) Example of normalized Gaussian curve used to calculate the angular distribution of the laminate for the *Crh^+/+^* bone. Its FWHM was determined from Fig. 2C. The area behind the central region of the curve is 74% of the total area. As a result, we adopted an angular distribution with 74% of 0°, 24% of ±30° and 2% of ±60° laminae (considering right and left areas of the Gaussian curve). Analogous process led to the following angular distribution for the GIOP case: 64% of 0°, 30% of ±30° and 6% of ±60° laminae. E) By fitting the analytical model with the experimental data (see also Fig. 5A) the aspect ratios (AR) for the *Crh^+/+^* and Crh*^−120/+^* bones were calculated (respectively, 15.0 for the *Crh^+/+^* case and 9.6 for the Crh*^−120/+^* case).

We modelled the lamellar structure of bone as layers of mineralized fibrils. Specifically, at the nanometre scale, the mineralized fibrils are made of mineral platelets that are embedded in a collagen matrix (both materials mechanically isotropic) and arranged in a staggered configuration, similar to previously described nanoscale models [52], [53], [54], [55]. These fibrils form, at a higher hierarchical level (micro-scale), a plywood lamellar system, also known as a Bouligand or rotated plywood structure [56], made of several layers (sub-lamellae or laminae, in engineering terms [57]) arranged as a stack and mechanically orthotropic (Fig. 2A). Specifically, the thickness of the lamellar structure is 10 μm while the thickness of each of the 100 sub-layers forming the structure is 100 nm.

The effective mechanical properties at the lamellar level were calculated using lamination theory for composites [57]. In detail, the modulus of each lamina (or sub-lamella) along the fibril direction (*E*_1_) was obtained by homogenizing the material properties of the fibril using the constitutive relation for mineralized fibrils [55]. This relation takes into account the staggered arrangement of mineral platelets, their aspect ratio (AR) and their volume fraction. For the laminar modulus *E*_2_ transverse to the fibril, and the laminar shear modulus *G_12_*, the Reuss model (fibres and matrix arranged in layers perpendicular to loading direction) was used. Lastly, the laminar Poisson’s ratios were calculated by adopting the Voigt rule of mixtures. All the material parameters as well as the relevant equations at the laminar level implemented are included in the Supplementary Information. Based on these input parameters, the longitudinal elastic modulus of the entire lamella (along the 0° sublamella) was calculated from standard lamination theory [57]. This parameter is compared against the effective fibril modulus E_f_ obtained from the *in situ* SAXD experiments.

The discretization provided by the plywood model in [58] (set of laminae of different thickness and angularly separated by 30°) was adopted in order to model the angular arrangement of laminae (Fig. 2B). The lamination sequence of the plywood system was calculated through a three-step process. Firstly, for a series of simulated azimuthal *I*(χ) plots with increasing FWHM (full width at half maximum), the ρ-parameter (described in Section 2.5) was calculated and plotted versus FWHM (Fig. 2C). Secondly, from the experimentally obtained ρ-parameters (calculated as in 2.5), an average value was chosen for each bone-type, and the equivalent FWHM calculated. Normalized Gaussian curves with these FWHM’s were calculated for each bone type (Fig. 2D shows a curve for *Crh^+/+^*). By discretizing the Gaussian curve, as shown in Fig. 2D, the relative proportions of fibrils at 0°, ±30° and ± 60° were obtained. These lamellar structural parameters were input into the lamination theory model, and the numerical results were fitted to the experimental data by varying the aspect ratio AR of the mineral particles in both cases.

## 3 Results

### 3.1 *In-situ* tensile testing with synchrotron SAXD

**Fig. 3:**
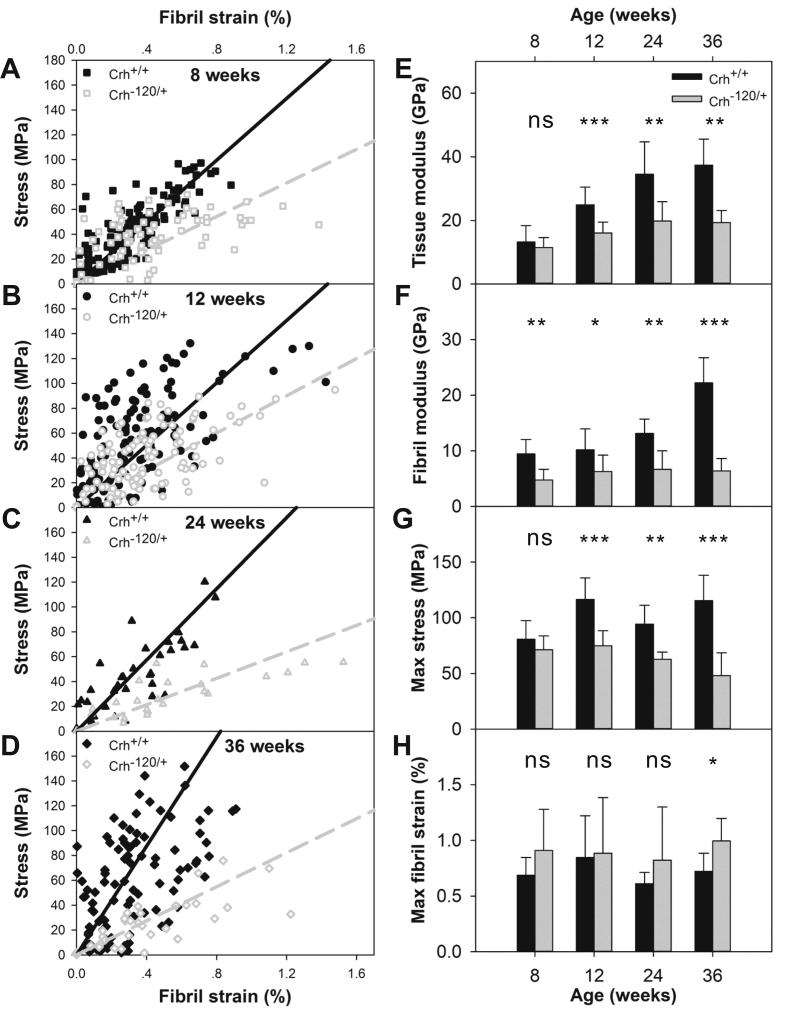
Tissue and fibrillar level mechanics: (A–D) Tissue level stress plotted as a function of fibril strain for femoral cortical bone from *Crh*^−120/+^ and *Crh^+/+^* mice aged (A) 8 weeks, (B) 12 weeks, (C) 24 weeks and (D) 36 weeks. Straight lines are linear regressions (solid black: *Crh^+/+^*; dashed gray: *Crh^−120/+^*) (E) Tissue level modulus (GPa) plotted as a function of genotype and developmental age. (F) Effective fibril modulus (GPa) plotted as a function of genotype and developmental age. (G) Maximum stress (MPa) plotted as a function of genotype and developmental age. (H) Maximum fibril strain (%) plotted as a function of genotype and developmental age. Error bars shown are standard deviations. Student’s *t*-tests were performed to determine if there are statistical significance in the differences between effective fibril modulus, maximum fibril strain, tissue modulus, and maximum stress between *Crh^+/+^* and *Crh*^−120/+^ mice at four developmental ages. Statistical significance is denoted (*p < 0.05, **p < 0.01, ***p < 0.001, ns: not significant).

Typical stress-tissue strain and fibril strain – tissue strain plots for femoral mid-shaft of *Crh^+/+^* and *Crh*^−120/+^ mice aged 24 weeks are shown in Fig. 1E, F. Considering deformation at the fibrillar level, the combined data sets for macroscopic stress versus nanoscale fibrillar strain (Fig. 3A–D) in the elastic deformation region for both *Crh*^−120/+^ and *Crh^+/+^* mice at each age points revealed differences in the slope, which was defined as the effective fibril modulus (E_f_ = dσ/dε_f_). All samples with the same genotype and developmental age were plotted together using the same symbol. Black solid and gray hollow scattering symbols represent *Crh^+/+^* and *Crh*^−120/+^ mice, respectively. Black and gray straight lines indicate regressions of all data points in each group for *Crh^+/+^* and *Crh*^−120/+^ mice, respectively.

The average effective fibril modulus was plotted as a function of genotype and age, as shown in Fig. 3F. As age increased from 8 to 36 weeks, the effective fibril modulus increased by 12.7 GPa (from 9.5 to 22.2 GPa) in *Crh^+/+^* mice, whereas it only increased by 1.6 GPa (from 4.8 to 6.4 GPa) in *Crh*^−120/+^ mice. At each age point, the effective fibril modulus was significantly lower in *Crh*^−120/+^ mice as compared with *Crh^+/+^* mice. There is a statistically significant difference (*p* < 0.001) among 4 ages for effective fibril modulus in *Crh*^+/+^ mice, but none in *Crh*^−120/+^ mice. In *Crh*^+/+^ mice, the significant differences are between fibril modulus at 36 weeks versus fibril modulus at 8, 12 and 24 weeks (Table S3, Supplementary Information). The maximum fibrillar strain was significantly higher in *Crh*^−120/+^ mice compared to *Crh^+/+^* mice at 36 weeks (*p* < 0.05), but not at other age points (Fig. 3H).

Tissue level modulus as a function of genotype and age was plotted in Fig. 3E. Tissue modulus were lower in *Crh*^−120/+^ bone compared to *Crh^+/+^* bone, with significant difference for mice at 12 (*p* < 0.001), 24 (*p* < 0.01) and 36 weeks (*p* < 0.01), but not for mice at 8 weeks (*p* > 0.05). There is a statistically significant difference in tissue modulus across 4 ages in both *Crh*^+/+^ (*p* < 0.001) and *Crh*^−120/+^ mice (*p* < 0.01). In *Crh*^+/+^ mice, tissue modulus at 36 weeks is significantly different from tissue modulus at 8 and 12 weeks, and tissue modulus at 8 weeks is significantly different from tissue modulus at 24 weeks. In *Crh*^−120/+^ mice, tissue modulus at 8 weeks is significantly different from tissue modulus at 24 and 36 weeks. For further details, refer to Table S4, Supplementary Information. Maximum stresses were significantly higher in *Crh^+/+^* bone compared to *Crh*^−120/+^ bone at 12 weeks (*p* < 0.001), 24 weeks (*p* < 0.01) and 36 weeks (*p* < 0.001), while the difference was not significant at 8 weeks (Fig. 3G).

### 3.2 BSE imaging and X-ray microtomography

**Fig. 4:**
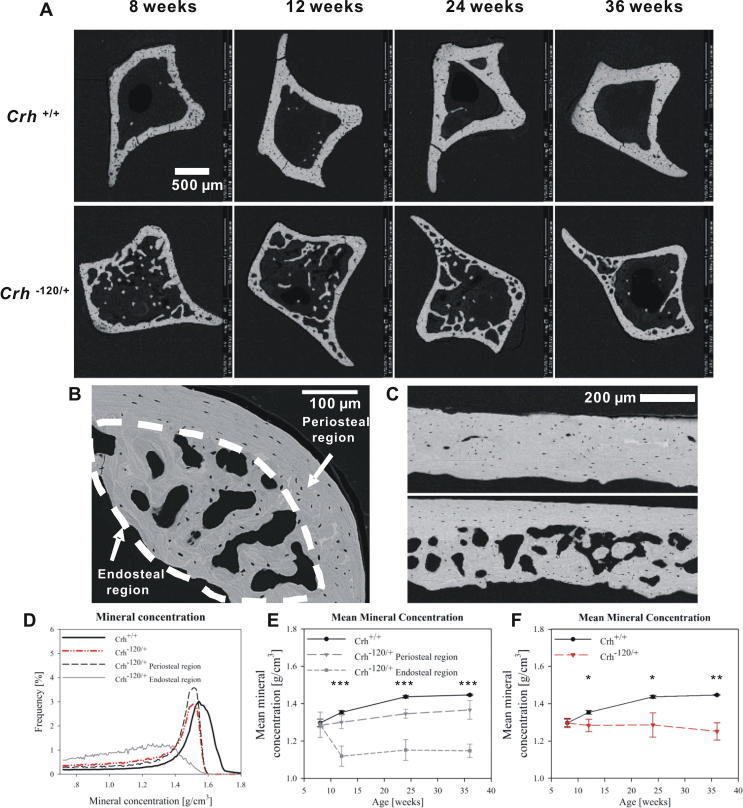
Tissue mineralization: (A) BSE images of transverse cross sections of tibia mid-diaphysis from *Crh^+/+^* and *Crh*^−120/+^ mice bone with different ages (8, 12, 24 and 36 weeks). (B) Transverse cross section of mid-shaft femora from *Crh*^−120/+^ mice aged 24 weeks. Periosteal region and endosteal region with halos (dashed line) are indicated. (C) BSE image of longitudinal section of mid-shaft femora from *Crh^+/+^* (upper) and *Crh*^−120/+^ mice (lower) aged 24 weeks. (D) Histograms of mineral concentration obtained from X-ray tomography images of transverse cross section of mid-shaft femora from *Crh^+/+^*, *Crh*^−120/+^ bone, and *Crh*^−120/+^ periosteal and endosteal regions aged 24 weeks. (E) Mean mineral concentration as a function of developmental ages for *Crh^+/+^*, *Crh*^−120/+^ periosteal regions and *Crh*^−120/+^ endosteal regions. (F) Mean mineral concentration as a function of development age (weeks) for *Crh^+/+^* (black circles) and *Crh*^−120/+^ mice (red triangles (colour online), including both periosteal and endosteal regions). Error bars shown are standard deviations. Statistical significance is denoted (*p < 0.05, **p < 0.01, ***p < 0.001, ns: not significant). (For interpretation of the references to colour in this figure legend, the reader is referred to the web version of this article.)

BSE images (Fig. 4A) were acquired on polished cross sections of tibia mid-diaphysis to examine possible mineralization defects in *Crh*^−120/+^ mice. A striking difference in cortical microstructure between the *Crh*^−120/+^ and *Crh^+/+^* mice was observed at all four ages. As reported in prior histochemical studies on this model [34], the endosteal surface for *Crh*^−120/+^ mice has a surface which has ruffled and porous characteristics with cavities, resembling cancellous bone, which is believed to arise from multiple factors: reduced osteoblast coverage on the cortico-endosteal surface, lower number of osteoblasts and slower mineralizing rate. The overall cortical thickness of *Crh*^−120/+^ mice was smaller than *Crh^+/+^* mice, and the cross sections of *Crh*^−120/+^ tibia had a very large fraction of cavities, particularly near the endosteal cortex, leading to an increased porosity. We note also that the distribution of cavities around the circumference of the tibiae is not uniform. In contrast, *Crh^+/+^* tibia had a denser cortical cross-section and showed uniformly distributed lacunae around the full cortex. Fig. 4B shows a high magnification of transverse cross section of mid-shaft femora from *Crh*^−120/+^ mice aged 24 weeks, with periosteal and endosteal region indicated on the figure. A BSE image of longitudinal section of mid-shaft femora from *Crh^+/+^* (upper) and *Crh*^−120/+^ mice (lower) aged 24 weeks are shown in Fig. 4C. Again, there are many large cavities near the endosteal cortex in *Crh*^−120/+^ femora, whereas *Crh^+/+^* femora showed uniformly distributed lacunae and no such cavities.

X-ray microtomography was used to measure the three-dimensional morphology and mineral concentration of mid-shaft femora. Microtomography of the mid-shaft femora used for the *in situ* SAXD testing were used, rather than the tibial sections used for BSE in Fig. 4A, in order to obtain the mineral concentration of samples where fibril moduli has been determined, and to avoid measurement errors from the angular anisotropy of the endosteal cancellous-like structure. Fig. 4D shows plots of the tissue mineral concentration obtained from X-ray tomography images of transverse cross sections of mid-shaft femora from *Crh^+/+^*, *Crh*^−120/+^ bone, and *Crh*^−120/+^ periosteal and endosteal regions aged 24 weeks. We note that the mineral concentration refers to the bone tissue alone, excluding all voids and spaces within the tissue.

The mean mineral concentrations (from X-ray microtomography measurements) were plotted as a function of developmental age for *Crh^+/+^* mice, periosteal regions away from cavities and endosteal regions near cavities in *Crh*^−120/+^ mice (Fig. 4E). Regarding the data in Fig. 4E, one-way ANOVA tests showed that the mean mineral concentration in *Crh^+/+^* mice, periosteal (*Crh*^−120/+^*-PR*) and endosteal regions (*Crh*^−120/+^*-ER*) in *Crh*^−120/+^ mice were similar at 8 weeks and the difference is not significant (*p* = 0.922). After 8 weeks, however, there are significant (*p* < 0.001) differences of the mean mineral concentrations between these three groups (*Crh^+/+^*, *Crh*^−120/+^*-PR*, *Crh*^−120/+^*-ER*) at 12, 24 and 36 weeks (Table S2, Supplementary Information). As can be seen from this table together with Fig. 4E, post-hoc tests showed that at 12 weeks, *Crh*^−120/+^*-ER* was significantly lower than both *Crh*^−120/+^*-PR* (*p* = 0.003) and *Crh^+/+^* (*p* < 0.001). Similarly, at 24 weeks, *Crh*^−120/+^*-ER* was significantly lower than both *Crh*^−120/+^*-PR* (*p* = 0.001) and *Crh^+/+^* (*p* < 0.001), and at this age-point the difference between *Crh*^−120/+^*-PR* and *Crh^+/+^* was also significant (*p* = 0.045). Lastly, at 36 weeks, *Crh*^−120/+^*-ER* was also significantly lower than both *Crh*^−120/+^*-PR* (*p* < 0.001) and *Crh^+/+^* (*p* < 0.001).

It is observed that the rate of increase in mineralization with age was generally greater in *Crh^+/+^* mice than in the *Crh*^−120/+^*-PR* regions in *Crh^−^*^120/+^ mice, whereas the mean mineral concentration decreased dramatically from ∼1.68 g/cm^3^ at 8 weeks to ∼1.46 g/cm^3^ at 12 weeks and kept nearly constant after 12 weeks in the *Crh*^−120/+^*-ER* endosteal regions in *Crh*^−120/+^ mice. Indeed, one-way ANOVA tests shows that the variation with age was significant (*p* < 0.001) for *Crh*^+/+^, significant (*p* = 0.021) for the endosteal region of *Crh*^−120/+^ (*Crh*^−120/+^*-ER*), and nonsignificant (*p* = 0.053) for the periosteal region of *Crh*^−120/+^ (*Crh*^−120/+^*-PR*). Considering the *Crh*^−120/+^*-ER* zone, Tukey’s post-hoc tests show (Table S1, Supplementary Information) that the significant (*p* = 0.022) difference is the drop between 8 and 12 weeks, in line with the qualitative observation from BSE images that there are less halos and hypomineralized regions in *Crh*^−120/+^ endosteal regions at 8 weeks compared to later time-points. For the *Crh*^+/+^ mice bone, Tukey’s post-hoc tests (Table S1) show significant (*p* < 0.05) differences between all ages, except between the last two age points (24 versus 36 weeks), characteristic of an initial rise in mineralization followed by a levelling-off. These variations across four developmental ages showed that the mineral concentration was impaired over age in *Crh*^−120/+^ mice as compared with *Crh^+/+^* mice, specifically in the cancellous-like tissue in the endosteal regions in *Crh*^−120/+^ mice.

Lastly, when the periosteal and endosteal regions are not separately considered, and an average mineral concentration calculated for *Crh*^−120/+^ mice, Fig. 4F shows the mean mineral concentration as a function of development age (weeks) for *Crh*^+/+^ and *Crh*^−120/+^ mice. In this case, Student’s *t*-tests showed significant differences in the mean mineral concentrations between *Crh^+/+^* and *Crh*^−120/+^ mice at 12, 24 and 36 weeks. There is not a statistically significant difference (*p* = 0.655) in the mean mineral concentrations across the 4 ages in *Crh*^−120/+^ bone as a whole.

### 3.3 Model results

The model described in Section 2.11 was used to compare the effective fibril modulus (experimentally calculated) with the longitudinal (along the direction of 0° laminae) stiffness of the whole laminated (numerically calculated *via* lamination theory [57]). As described in 2.11, using an average value of ρ of 0.45 for *Crh^+/+^* bone and 0.3 for the *Crh*^−120/+^ bone (from Fig. 6D), the equivalent FWHM for each bone type was calculated to be 48° (*Crh^+/+^*) and 57° (*Crh^−120/+^*) respectively. By discretizing the equivalent normalized Gaussian curve, we obtain the relative proportions of fibrils in different laminae (sublamellae) in the Bouligand structure. For *Crh^+/+^*, we obtained 74% of 0° laminae, 24% of ±30° laminae and 2% of ±60° laminae, whilst (as expected) for *Crh^−120/+^* bone a broader Gaussian distribution of 64% of 0° laminae, 30% of ±30° laminae and 6% of ±60° laminae were found. Fig. 2D shows the discretization for the case of *Crh^+/+^* bone.

**Fig. 5:**
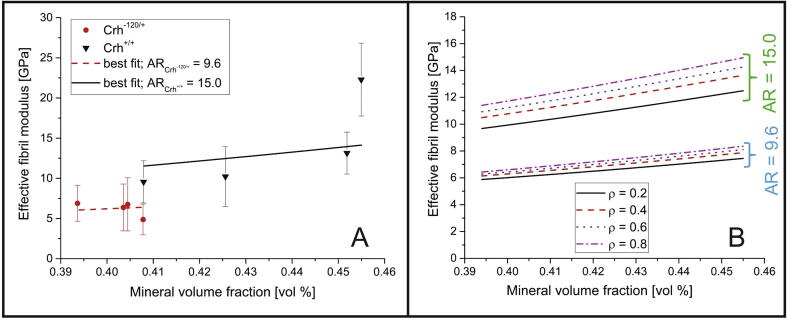
Model results: A) Comparison between experimental data and numerical best fit curves. The bars indicate the standard deviation of experimental results. From the best fit process two different values of AR (aspect ratio) were calculated: 15.0 for the *Crh^+/+^* and 9.6 for the *Crh*^−120/+^ case. B) Effective fibril modulus vs mineral volume fraction as function of the aspect ratio AR of mineral platelets and ρ (level of alignment of mineral particles).

Numerical curves (Fig. 5A) were obtained by minimizing the mean squared difference between the experimental E_f_ values (at the measured values of mineral volume fraction) and the model predictions for E_f_. This best-fit process allowed us to estimate the aspect ratio AR of mineral particles within mineralized fibrils in *Crh^+/+^* and *Crh*^−120/+^ bone (15.0 and 9.6, respectively), as shown schematically in Fig. 2E. The quality of the best fit was assessed by a chi-squared test of the residual sum of squares (normalized by the standard deviations) with χ^2^ reaching minimal values at 15.0 (χ^2^ = 4.36) and 9.6 (χ^2^ = 0.82), respectively, for *Crh^+/+^* and *Crh^−120/+^* bone. The larger χ^2^ for *Crh^+/+^* reflects the fact that the 36-week experimental value lies well above the fit line.

We then used the model to create parametric maps for the fibril modulus of bone (Fig. 5B), where the effective fibril modulus of interest is determined for a certain range of the mineral volume fraction and several values of mineral AR- and ρ-parameter values. This graph could, in future, be used for an inverse calculation in case the AR of mineral platelets and the ρ-parameter are known and the effective fibril modulus and the mineral volume fraction are desired as output.

### 3.4 L-parameter and lattice spacing of the mineral crystallites and ρ-parameter

**Fig. 6:**
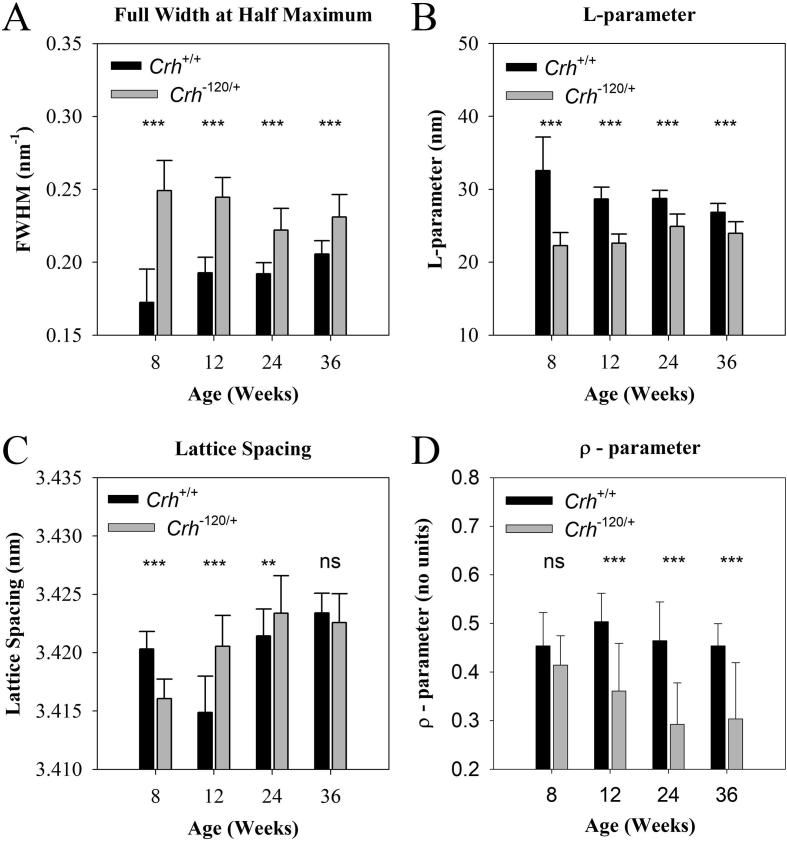
Nanoscale structural parameters of bone mineral and fibrils: (A) FWHM of the (0 0 2) reflection of HA as a function of age for *Crh^+/+^* and *Crh^−120/+^* bone at different ages. (B) L-parameter and (C) lattice spacing of HA crystallite for *Crh^+/+^* and *Crh^−120/+^* bone at different ages. (D) Degree of alignment of the mineral crystals (ρ-parameter) as a function of age and disease condition. Error bars shown are standard deviations. Statistical significance is denoted (*p < 0.05, **p < 0.01, ***p < 0.001, ns: not significant).

Motivated by the difference in AR – between *Crh^+/+^* and *Crh^−120/+^* bone – predicted by the model, we analysed wide-angle X-ray diffraction (WAXD) patterns on the bone types acquired with the microfocus beam. We wished to determine if there was experimental evidence for the lowered AR of the mineral crystallites. Fig. S2 (Supplementary Information) shows typical 2D profiles of the (0 0 2) reflection of HA in femur mid-diaphysis from *Crh^+/+^* and *Crh^−120/+^* mice bone aged 12 weeks. The (0 0 2) reflections were fitted with a Lorentzian profile to obtain the FWHM. When comparing groups at each age point, as shown in Fig. 6A the FWHM in *Crh^−120/+^* mouse bone is significantly higher than in *Crh^+/+^* mouse bone at all ages (*p* < 0.001). Correspondingly the L-parameter in *Crh^−120/+^* mouse bone is significantly lower than in *Crh^+/+^* mouse bone at all ages (Fig. 6B, *p* < 0.001). Finally, the lattice spacing shows an overall increasing – but non-monotonic – trend for both groups (Fig. 6C).

One-way ANOVA tests shows that there is a statistically significant variation of the three parameters – L-parameter, FWHM, and lattice spacing – from the (0 0 2) profiles across age-points in both *Crh*^+/+^ and *Crh^−120/+^* groups (Tables S5–S7, Supplementary Information). The difference is significant (*p* < 0.001) for all six combinations (3 parameters and 2 bone types). Regarding the L-parameter, it exhibits a decreasing tendency with age for the healthy *Crh^+/+^* mouse, and an increasing trend for the *Crh^−120/+^* mouse. Post-hoc Tukey tests reveal (Table S7) that for *Crh^−120/+^* mouse bone, the first two age points are different from the last two: specifically, there are significant differences (*p* < 0.001) between 8- and 24-weeks, and between 12- and 24-weeks, and significant (*p* = 0.002) differences between 8- and 36 weeks, and between 12- and 36 weeks. For the healthy *Crh^+/+^* mouse bone, differences between 8-weeks and all other age-points are significant (*p* < 0.001). The FWHM, being proportional to the inverse of the L-parameter (Materials and Methods), follows a similar pattern (Table S7). For the (0 0 2) lattice parameter, post-hoc Tukey tests show (Table S7) for *Crh^−120/+^* mouse bone a significant (*p* < 0.001) difference between 8-weeks and all other age-points, a significant (*p* < 0.001) difference between 12- and 24-weeks and a significant (*p* = 0.014) difference between 12- and 36-weeks. The corresponding post-hoc tests in the healthy *Crh^+/+^* mouse bone find significant differences (*p* < 0.001) between 8-weeks and both 12- and 36-weeks, as well as between 12-weeks and both 24- and 36-weeks, and significant (*p* = 0.005) differences between 24- and 36-weeks.

Lastly, ρ-parameters were plotted as a function of age and disease condition in Fig. 6D. Student’s *t*-tests showed that ρ-parameters are consistently significantly higher (*p* < 0.001) in *Crh^+/+^* than in *Crh^−120/+^* mice at age 12, 24 and 36 weeks, whereas there is no significant difference between them at 8 weeks. Regarding age-variation of the ρ-parameter, there is a monotonic decreasing trend in *Crh^−120/+^* mice, which is significant (*p* < 0.001) across ages (Table S8, Supplementary Information). Post-hoc testing shows, for *Crh^−120/+^* mice, significant (*p* < 0.001) differences between 8-weeks and both 24- and 36-weeks, and significant differences (*p* = 0.027) between 12- and 24-weeks. Although the ρ-parameter for *Crh^+/+^* mice shows no monotonic trend, there are also very significant (*p* = 0.002) differences between ages. In this case, the 12-week point is significantly (*p* = 0.005) higher than the 8-week values, and significantly (*p* = 0.002) higher than the subsequently decreased values at 36-weeks.

## 4 Discussion

In summary, our main findings are:

• Consistently reduced fibrillar modulus in *Crh^−120/+^* mouse bone in comparison to *Crh^+/+^* mouse bone, with minimal increase in fibrillar modulus with age in *Crh^−120/+^* mouse bone, in contrast to an over 2-fold increase in *Crh^+/+^* mouse bone fibril modulus (Fig. 3).
• A heterogeneous and lower mineralization density distribution at the microscale, accompanied by a more porous 3D microarchitecture in *Crh^−120/+^* mouse bone (Fig. 4).
• A laminate fibril array model – based on the rotated plywood lamella [56], [59], and deriving its parameters from experimental structural data – explains the reduced effective fibril modulus (measured experimentally) in *Crh^−120/+^* mouse bone in comparison to *Crh^+/+^* mouse bone (Fig. 5).
• In terms of the model parameters, the main difference between *Crh^−120/+^* and *Crh^+/+^* is a reduced mineral platelet aspect ratio AR (15.0 (*Crh^+/+^*) to 9.6 (*Crh^−120/+^*)) at the scale of the mineralized collagen fibril (Fig. 2, Fig. 5).
• Reduced length of mineral platelets in *Crh^−120/+^* bone, as measured from the Debye-Scherrer width, consistent with the reduced AR predicted by the model (Fig. 6).
• A greater degree of fibrillar disorganization (measured via the ρ-parameter) in *Crh^−120/+^* bone compared to *Crh^+/+^* (Fig. 6).

In terms of our hypothesis about alterations in the fibrillar structure-function relations, the results provide clear evidence for changes in fibrillar deformation mechanisms in *Crh^−120/+^* compared to *Crh^+/+^*, which are consistent throughout development (see, e.g. Fig. 3F). Concurrently, the degree of fibrillar disorganization (given by the ρ-parameter) shows similar values between *Crh^+/+^* and *Crh^−120/+^* at the earliest time-point investigated (8 weeks) but a rapid decrease in the ρ-parameter in *Crh^−120/+^* relative to *Crh^+/+^* for all subsequent age-points (Fig. 6D). The experimental (Fig. 3, Fig. 4) and modelling (Fig. 5) results on the fibrillar deformation provide evidence that the lowered overall mineralization may also have an additional mechanically significant effect at the fibrillar level, through the reinforcement of the collagen fibril *via* the staggered arrangement of mineral and collagen. Therefore, while bone microarchitecture and overall mineralization level is important for the mechanical alteration in *Crh^−120/+^*, so also is the nanocomposite architecture.

The mineralized microstructure in *Crh^−120/+^* (Fig. 4A–C) shows a distinct region on the endosteal side, resembling cancellous bone, where lower overall mineral density and a porous microstructure are found. Together with the structural normality of the periosteal region, these results suggest the mechanistic effects in cortical bone are solely due to this endosteal hypomineralized zone. Our findings of a lowered L-parameter, and a reduced aspect ratio AR in the model, makes it clear that the ultrastructural bone mineral shape, as well as its overall volume fraction, is mechanically important both at the nano- and macro-length scales. This is due to the shorter mineral platelets in *Crh^−120/+^* (lower AR) having a reduced mechanical reinforcing efficiency in the collagen matrix. Prior simulation and experimental work on the mineralized collagen fibril have emphasized the importance of the mineral platelet structural anisotropy (e.g. [17], [52], [54], [55]).

This heterogeneous mineralized matrix structure is likely created by the alteration in cellular activity in GIOP [1]. Consistent with prior work on osteoclast activation, osteoblast suppression and trench-like cavities formed in GIOP bone [10], [25], the endosteal cortical bone in the *Crh*^−120/+^ model exhibits extensive excavation of elongated cavities. The mineralization in the endosteal region is, on average, characteristic of less mature tissue and even, quantitatively, to hypomineralized conditions like osteomalacia [60]. It is also possible, although this cannot be confirmed with the current data alone, that the proposed “osteolytic osteolysis” mechanism is involved in the overall lower mineralization, due to leaching of mineral from around osteocyte lacunae [61]. Our prior high magnification electron microscopy images on *Crh^−120/+^* mouse bone [32] showed haloes of lower mineralized tissue around the porous endosteal tissue. Future scanning microbeam X-ray scattering and diffraction measurements around such cavities may shed light on the dynamics of mineralized tissue formation in *Crh^−120/+^*.

The model of the mineralized fibrils presented here, containing anisometric mineral platelets, highlights the importance of the nanoscale architecture in the stiffness. The multiscale model presented here is similar to the fibril/lamellar models presented earlier for bone (e.g. [52], [54], [55], [62], [63], [64]). At the scale of the fibril, the deformation in the bone matrix operates *via* a shearing in the collagen layers combined with a mainly tensile load in the mineral platelets. Load transfer between collagen and mineral is facilitated by the long aspect ratio of the platelets, resulting in large interfacial area of contact between the two phases for shear transfer. As a result, beyond a simple rule of mixtures for the mineral and collagen, a staggered model of the mineralized fibrils means that the overall tissue modulus depends on AR *via* Eqs. (5) and (9) in the Supplementary Information [55]. Therefore, variations in AR will have considerable effects on the modulus. It is assumed that the interfibrillar matrix contributes negligibly to the overall stress here (due to its very small volume fraction ∼2–5%[17], [55]). Hence, the stress on the fibrillar-phase in tension is the majority of stress on the bone matrix (σ_T_ = Φ_1_ σ_F_ + (1 − Φ_1_) σ_IF_ – σ_F_; σ_T_ tissue stress, σ_F_ fibril stress; σ_IF_ interfibrillar matrix stress, and Φ_1_ – 1 is fibril volume fraction). As a result, the fibril modulus – under these assumptions – is a good proxy for the tissue modulus at the material level (after correction for microscale porosity). In relation to prior multiscale staggered models (e.g. [52], [53], [54], [55], [62], [63]), in the present work we incorporate, from experimental data, structural parameters from motifs like the plywood structure (such as their angular widths). The second is the comparison of deformation mechanisms between model and experiment at multiple scales rather than at the macroscopic tissue level only.

Some technical limitations and assumptions of the current work are noted. Due to the transmission nature of the SAXD/WAXD measurements, all nano- and molecular-level information is integrated across a scattering volume in the form of a tunnel with diameter ∼10 μm and length ∼200 μm going through the periosteal and endosteal regions. This means that the lowered fibrillar modulus and increased WAXD peak width is a weighted average of ultrastructural information from the endosteal region (lowered mineralization) and the periosteal region (Fig. 4B). Therefore, the actual ultrastructure in the bone matrix region most affected (endosteal region) is likely to be even more deviant from healthy bone. Secondly, the porous microarchitecture is not integrated fully into the multiscale model, and appears as a correction to the stress only. The application of multiscale models including porosity at different levels, as in [65], would be beneficial in this regard. Finally, it was assumed that the basic building block of the microstructural level (the lamella) is similar between *Crh^−120/+^* and *Crh^+/+^*, and only distributed differently across the tissue zones.

In conclusion, combined ultrastructural nanomechanical experiments and modelling analysis have been carried out to determine the mechanical differences between murine bone with glucocorticoid-induced osteoporosis and healthy bone, as a function of tissue age and development. It is suggested that the combination of an altered mineral nanostructure together with overall lower mineralization and fibrillar disorganization is the major factor behind both the lowered fibrillar mechanical properties and the lack of increase of fibrillar modulus with age in *Crh^−120/+^*. The combination of fibrillar-level deformation *via* X-ray diffraction, together with analytical modelling, helps to provide a self-consistent check of how the effects of alterations at the nano- and microscale propagate up the levels of structural hierarchy in bone. Such an approach will be useful in understanding the relevant nanostructural changes, mediated by biological activity, that alter or cause deterioration in musculoskeletal function in bone and cartilage disorders.
