# Supplementary material for: Bone matrix development in steroid-induced osteoporosis is associated with a consistently reduced fibrillar stiffness linked to altered bone mineral quality
Source: Acta Biomater. 2018 Aug;76:295–307. doi: 10.1016/j.actbio.2018.05.053 (PMC6084282; doi:10.1016/j.actbio.2018.05.053)
Supplement: Supplementary data 1 [file mmc1.docx]

**Supplementary Information**:

**S1: Analysis of microscale porosity**:


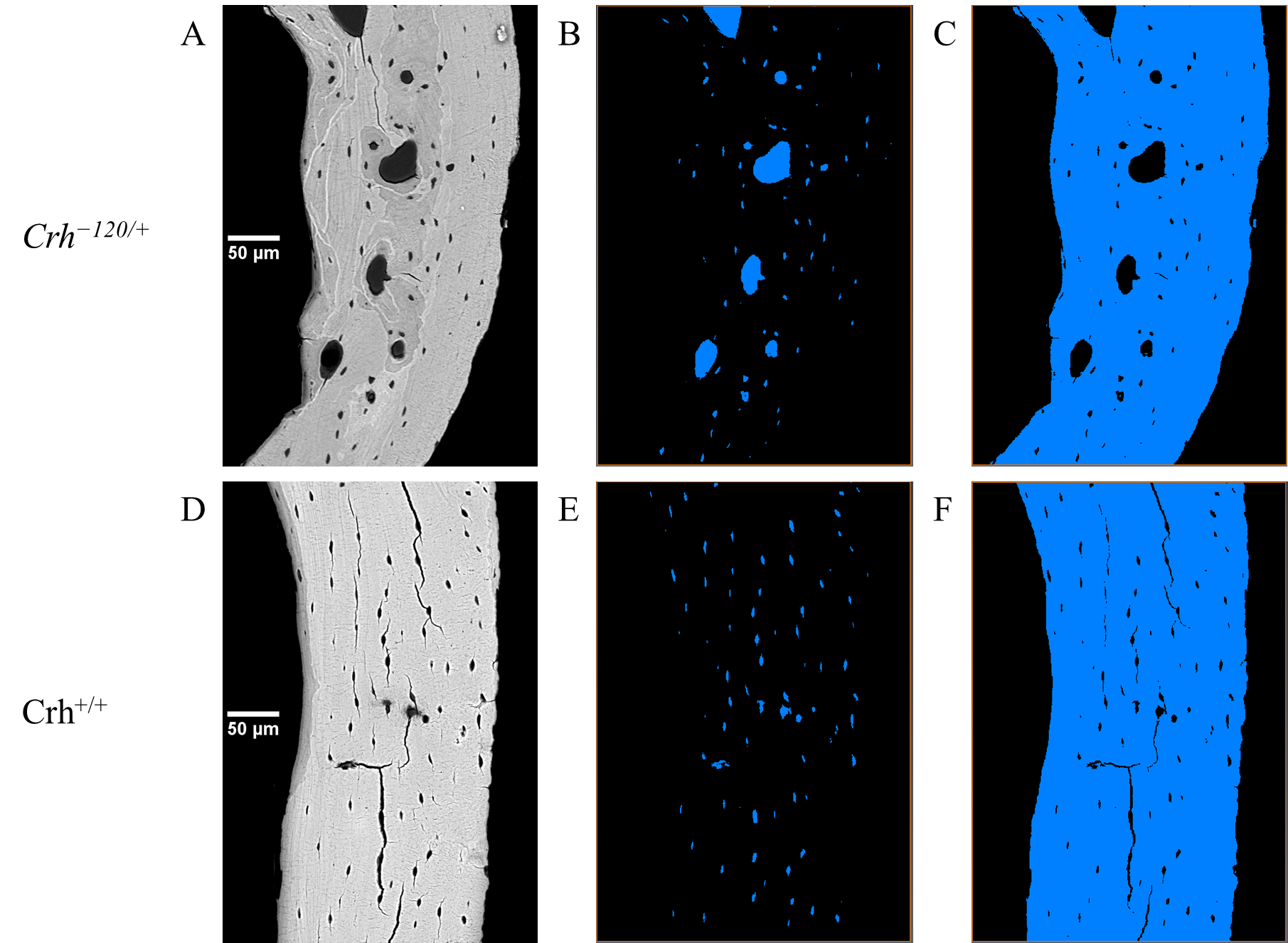


***Figure S1****:* ***Analysis of bone porosity****. (A) and (D) are BSE images (gray level 0-255) of transverse section from femur mid-diaphysis of Crh^−120/+^ and* Crh^+/+^ *mouse, respectively, aged 36 weeks. (B) and (E) are segmentations of voids (blue) from bone tissue with gray level between 0 and 60. Cracks are removed during segmentation of voids. (C) and (F) are segmentations of bone tissue (blue) with gray level between 60 and 255.*

**S2: 2D profiles of the (002) reflection of HA in femur**


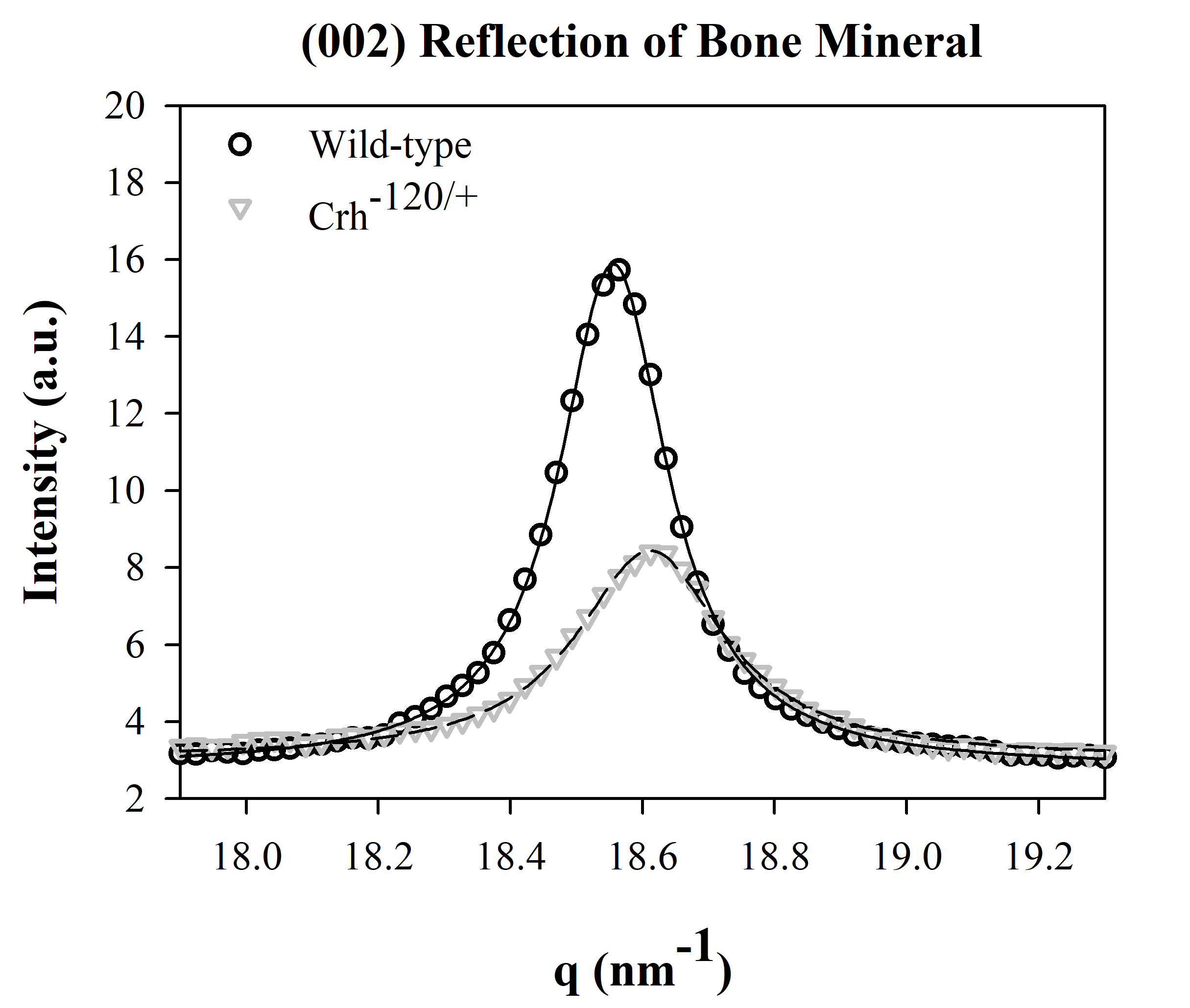


**Figure S2**: Typical 2D profiles of the (002) reflection of HA in femur mid-diaphysis from Crh^+/+^ and Crh^-120/+^ bone aged 12 weeks. Solid lines are fitting curves based on a Lorentzian profile.

**S3: Equations for multiscale mechanical model**:

The moduli of the individual sub-lamellae (laminae) are reported here. The relations below refer to the mineralized collagen fibril. φ_m_ is the mineral volume fraction, *E* is the Young’s modulus while *G* is the shear modulus. The subscripts *m* and *c* indicate respectively the mineral and collagen contents. The aspect ratio of the mineral platelet is AR. The symbol ‘/’ indicates that **Equation S1** can be used for the shear modulus of both, the mineral and collagen contents (under the assumption of isotropic material).

$$G_{m/c}=\frac{E_{m/c}}{2\left( 1+\nu_{m/c} \right)} (S1)$$

$$E_{1}=\frac{E_{m}\varphi_{m}}{k}+\left( 1-\varphi_{m} \right)E_{c} (S2)$$

$$E_{2}=\frac{{E_{m}E}_{c}}{\left( \varphi_{m}E_{c} \right)+\left( \left( 1-\varphi_{m} \right)E_{m} \right)} (S3)$$

$$G_{12}=\frac{{G_{m}G}_{c}}{\left( \varphi_{m}G_{c} \right)+\left( \left( 1-\varphi_{m} \right)G_{m} \right)} (S4)$$

$$\nu_{12}= \varphi_{m}\nu_{m}+\left( 1-\varphi_{m} \right)\nu_{c} (S5)$$

$$k=1+\left( \frac{4}{{AR}^{2}}\frac{1-\varphi_{m}}{\varphi_{m}}\frac{E_{m}}{\gamma_{c}E_{c}} \right) (S6)$$

The Young’s moduli (E) of the mineral and collagen constituents were 100 GPa and 2.5 GPa [[1](#_ENREF_1)] and their Poisson’s ratio (ν) was assumed to be 0.3. γ_c_ = 0.4 is a scaling factor which relates tensile modulus E_c_ to shear modulus [[1](#_ENREF_1)].

**ANOVA statistical tests and post-hoc tests**:

**Table S1: Mineral concentration across ages**:

(1) *Crh^+/+^*: Mean mineral concentration:

| Mean mineral concentration | | | <0.001 (***) | | |
| --- | --- | --- | --- | --- | --- |
| Age (weeks) | 8 | 12 | | 24 | 36 |
| 8 |  | 0.002 (**) | | <0.001 (***) | <0.001 (***) |
| 12 |  |  | | <0.001 (***) | <0.001 (***) |
| 24 |  |  | |  | 0.759 |
| 36 |  |  | |  |  |

(2) *Crh^-120/+^* Periosteal: Mean mineral concentration:

| Mean mineral concentration | | | 0.053 | | |
| --- | --- | --- | --- | --- | --- |
| Age (weeks) | 8 | 12 | | 24 | 36 |
| 8 |  | 0.929 | | 0.184 | 0.066 |
| 12 |  |  | | 0.396 | 0.152 |
| 24 |  |  | |  | 0.879 |
| 36 |  |  | |  |  |

(3) *Crh^-120/+^* Endosteal: Mean mineral concentration:

| Mean mineral concentration | | | 0.021 (*) | | |
| --- | --- | --- | --- | --- | --- |
| Age (weeks) | 8 | 12 | | 24 | 36 |
| 8 |  | 0.022 (*) | | 0.062 | 0.055 |
| 12 |  |  | | 0.878 | 0.913 |
| 24 |  |  | |  | 1.000 |
| 36 |  |  | |  |  |

(4) *Crh^-120/+^* Periosteal and Endosteal: Mean mineral concentration:

| Mean mineral concentration | | | 0.655 | | |
| --- | --- | --- | --- | --- | --- |
| Age (weeks) | 8 | 12 | | 24 | 36 |
| 8 |  | 0.982 | | 0.991 | 0.627 |
| 12 |  |  | | 1.000 | 0.825 |
| 24 |  |  | |  | 0.784 |
| 36 |  |  | |  |  |

**Table S2: Mineral concentration across bone regions**:

(1) 8 weeks mean mineral concentration:

| Mean mineral concentration | | 0.922 | |
| --- | --- | --- | --- |
| Region | *Crh^+/+^* | *Crh^-120/+^*-PR | *Crh^-120/+^*-ER |
| *Crh^+/+^* |  | 0.921 | 0.955 |
| *Crh^-120/+^*-PR |  |  | 0.995 |
| *Crh^-120/+^*-ER |  |  |  |

(2) 12 weeks mean mineral concentration:

| Mean mineral concentration | | <0.001 (***) | |
| --- | --- | --- | --- |
| Region | *Crh^+/+^* | *Crh^-120/+^*-PR | *Crh^-120/+^*-ER |
| *Crh^+/+^* |  | 0.268 | <0.001 (***) |
| *Crh^-120/+^*-PR |  |  | 0.003 (**) |
| *Crh^-120/+^*-ER |  |  |  |

(3) 24 weeks mean mineral concentration:

| Mean mineral concentration | | <0.001 (***) | |
| --- | --- | --- | --- |
| Region | *Crh^+/+^* | *Crh^-120/+^*-PR | *Crh^-120/+^*-ER |
| *Crh^+/+^* |  | 0.045(*) | <0.001 (***) |
| *Crh^-120/+^*-PR |  |  | 0.001 (**) |
| *Crh^-120/+^*-ER |  |  |  |

(4) 36 weeks mean mineral concentration:

| Mean mineral concentration | | <0.001 (***) | |
| --- | --- | --- | --- |
| Region | *Crh^+/+^* | *Crh^-120/+^*-PR | *Crh^-120/+^*-ER |
| *Crh^+/+^* |  | 0.073 | <0.001 (***) |
| *Crh^-120/+^*-PR |  |  | <0.001 (***) |
| *Crh^-120/+^*-ER |  |  |  |

**Table S3: Fibril modulus across ages**:

(1) *Crh^+/+^*: Fibril modulus:

| Fibril modulus | | | <0.001 (***) | | |
| --- | --- | --- | --- | --- | --- |
| Age (weeks) | 8 | 12 | | 24 | 36 |
| 8 |  | 0.981 | | 0.315 | <0.001 (***) |
| 12 |  |  | | 0.381 | <0.001 (***) |
| 24 |  |  | |  | <0.001 (***) |
| 36 |  |  | |  |  |

(2) *Crh^-120/+^*: Fibril modulus:

| Fibril modulus | | | 0.628 | | |
| --- | --- | --- | --- | --- | --- |
| Age (weeks) | 8 | 12 | | 24 | 36 |
| 8 |  | 0.703 | | 0.628 | 0.803 |
| 12 |  |  | | 0.993 | 1.000 |
| 24 |  |  | |  | 0.998 |
| 36 |  |  | |  |  |

**Table S4: Tissue modulus across ages**:

(1) *Crh^+/+^*: Tissue modulus:

| Tissue modulus | | | <0.001 (***) | | |
| --- | --- | --- | --- | --- | --- |
| Age (weeks) | 8 | 12 | | 24 | 36 |
| 8 |  | 0.049 (*) | | <0.001 (***) | <0.001 (***) |
| 12 |  |  | | 0.080 | 0.016 (*) |
| 24 |  |  | |  | 0.897 |
| 36 |  |  | |  |  |

(2) *Crh^-120/+^*: Tissue modulus:

| Tissue modulus | | | 0.010 (*) | | |
| --- | --- | --- | --- | --- | --- |
| Age (weeks) | 8 | 12 | | 24 | 36 |
| 8 |  | 0.180 | | 0.011 (*) | 0.038 (*) |
| 12 |  |  | | 0.310 | 0.559 |
| 24 |  |  | |  | 0.997 |
| 36 |  |  | |  |  |

**Table S5: (002) Lattice spacing across ages**:

(1) *Crh^+/+^*: lattice spacing:

| lattice spacing | | | <0.001 (***) | | |
| --- | --- | --- | --- | --- | --- |
| Age (weeks) | 8 | 12 | | 24 | 36 |
| 8 |  | <0.001 (***) | | 0.263 | <0.001 (***) |
| 12 |  |  | | <0.001 (***) | <0.001 (***) |
| 24 |  |  | |  | 0.005 (**) |
| 36 |  |  | |  |  |

(2) *Crh^-120/+^*: lattice spacing:

| lattice spacing | | | <0.001 (***) | | |
| --- | --- | --- | --- | --- | --- |
| Age (weeks) | 8 | 12 | | 24 | 36 |
| 8 |  | <0.001 (***) | | <0.001 (***) | <0.001 (***) |
| 12 |  |  | | <0.001 (***) | 0.014 (*) |
| 24 |  |  | |  | 0.684 |
| 36 |  |  | |  |  |

**Table S6: FWHM across ages**:

(1) *Crh^+/+^*: FWHM:

| FWHM | | | <0.001 (***) | | |
| --- | --- | --- | --- | --- | --- |
| Age (weeks) | 8 | 12 | | 24 | 36 |
| 8 |  | <0.001 (***) | | <0.001 (***) | <0.001 (***) |
| 12 |  |  | | 0.999 | <0.001 (***) |
| 24 |  |  | |  | 0.003 (**) |
| 36 |  |  | |  |  |

(2) *Crh^-120/+^*: FWHM:

| FWHM | | | <0.001 (***) | | |
| --- | --- | --- | --- | --- | --- |
| Age (weeks) | 8 | 12 | | 24 | 36 |
| 8 |  | 0.730 | | <0.001 (***) | 0.001 (**) |
| 12 |  |  | | <0.001 (***) | 0.005 (**) |
| 24 |  |  | |  | 0.173 |
| 36 |  |  | |  |  |

**Table S7: L-parameter across ages**:

(1) *Crh^+/+^*: L-parameter:

| L-parameter | | | <0.001 (***) | | |
| --- | --- | --- | --- | --- | --- |
| Age (weeks) | 8 | 12 | | 24 | 36 |
| 8 |  | <0.001 (***) | | <0.001 (***) | <0.001 (***) |
| 12 |  |  | | 1.000 | 0.014 (*) |
| 24 |  |  | |  | 0.049 (*) |
| 36 |  |  | |  |  |

(2) *Crh^-120/+^*: L-parameter:

| L-parameter | | | <0.001 (***) | | |
| --- | --- | --- | --- | --- | --- |
| Age (weeks) | 8 | 12 | | 24 | 36 |
| 8 |  | 0.864 | | <0.001 (***) | 0.002 (**) |
| 12 |  |  | | <0.001 (***) | 0.005 (**) |
| 24 |  |  | |  | 0.117 |
| 36 |  |  | |  |  |

**Table S8: ρ-parameter across ages**:

(1) *Crh^+/+^*: ρ-parameter:

| ρ-parameter | | | 0.002 (**) | | |
| --- | --- | --- | --- | --- | --- |
| Age (weeks) | 8 | 12 | | 24 | 36 |
| 8 |  | 0.005 (**) | | 0.937 | 1.000 |
| 12 |  |  | | 0.125 | 0.002 (**) |
| 24 |  |  | |  | 0.931 |
| 36 |  |  | |  |  |

(2) *Crh^-120/+^*: ρ-parameter:

| ρ-parameter | | | <0.001 (***) | | |
| --- | --- | --- | --- | --- | --- |
| Age (weeks) | 8 | 12 | | 24 | 36 |
| 8 |  | 0.196 | | <0.001 (***) | 0.001 (**) |
| 12 |  |  | | 0.027 (**) | 0.085 |
| 24 |  |  | |  | 0.971 |
| 36 |  |  | |  |  |

**References**

[1] Gupta H, Krauss S, Kerschnitzki M, Karunaratne A, Dunlop J, Barber A, Boesecke P, Funari S, Fratzl P. Intrafibrillar plasticity through mineral/collagen sliding is the dominant mechanism for the extreme toughness of antler bone. Journal of the Mechanical Behavior of Biomedical Materials 2013;28:366-82.
